# Ring chromosome 18 in combination with 18q12.1 (*DTNA)* interstitial microdeletion in a patient with multiple congenital defects

**DOI:** 10.1186/s13039-016-0229-9

**Published:** 2016-02-18

**Authors:** Anna Zlotina, Tatiana Nikulina, Natalia Yany, Olga Moiseeva, Tatiana Pervunina, Eugeny Grekhov, Anna Kostareva

**Affiliations:** Almazov Federal Medical Research Centre, Saint-Petersburg, 197341 Russia; Institute of translational Medicine, ITMO University, Saint-Petersburg, 199034 Russia; Department of Women’s and Children’s Health, Center for Molecular Medicine, Karolinska Institute, Stockholm, 17176 Sweden; Cytology and Histology Department, Saint Petersburg State University, Saint-Petersburg, 199034 Russia

**Keywords:** Array-based CGH, α-dystrobrevin, *DTNA* deletion, Ring(18), Subaortic stenosis

## Abstract

**Background:**

Ring chromosome 18 [r(18)] syndrome represents a relatively rare condition with a complex clinical picture including multiple congenital dysmorphia and varying degrees of mental retardation. The condition is cytogenetically characterized by a complete or mosaic form of ring chromosome 18, with ring formation being usually accompanied by the partial loss of both chromosomal arms. Here we observed a 20-year-old male patient who along with the features typical for r(18) carriers additionally manifested a severe congenital subaortic stenosis. To define the genetic basis of such a compound phenotype, standard cytogenetic and high-resolution molecular-cytogenetic analysis of the patient was performed.

****Case presentation**:**

Standard chromosome analysis of cultured lymphocytes confirmed 46, XY, r(18) karyotype. Array-based comparative genomic hybridization (array-CGH) allowed to define precisely the breakpoints of 18p and 18q terminal deletions, thus identifying the hemizygosity extent, and to reveal an additional duplication adjoining the breakpoint of the 18p deletion. Apart from the terminal imbalances, we found an interstitial microdeletion of 442 kb in size (18q12.1) that encompassed *DTNA* gene encoding α-dystrobrevin, a member of dystrophin-associated glycoprotein complex. While limited data on the role of *DTNA* missense mutations in pathogenesis of human cardiac abnormalities exist, a microdeletion corresponding to whole *DTNA* sequence and not involving other genes has not been earlier described.

**Conclusions:**

A detailed molecular-cytogenetic characterization of the patient with multiple congenital abnormalities enabled to unravel a combination of genetic defects, namely, a ring chromosome 18 with terminal imbalances and *DTNA* whole-gene deletion. We suggest that such combination could contribute to the complex phenotype. The findings obtained allow to extend the knowledge of the role of *DTNA* haploinsufficiency in congenital heart malformation, though further comprehensive functional studies are required.

**Electronic supplementary material:**

The online version of this article (doi:10.1186/s13039-016-0229-9) contains supplementary material, which is available to authorized users.

## Background

Ring chromosome 18 [r(18)] is a relatively rare structural chromosomal abnormality characterized by the replacement of a normal chromosome 18 by the ring chromosome. A ring formation is usually accompanied by the partial loss of both chromosomal arms that in turn leads to hemizygous state of genes from the deleted regions [1–3]. Patients bearing a r(18) have complex clinical presentation that combines phenotypic features of 18p (OMIM #146390) and 18q (OMIM #601808) deletion syndromes and includes multiple congenital defects with varying degrees of mental retardation [4, 5]. The most typical features include short stature, multiple facial dysmorphia such as a carp-shaped mouth, cleft lip/palate, broad flat nose and epicanthic folds, hypertelorism, hearing loss, different eye abnormalities, microcephaly, abnormal white matter, hypotonia, and, rarer, congenital heart defect most commonly in a form of pulmonary stenosis [2–6].

There is considerable phenotypic variability among patients with ring 18 that is mainly due to differences in extent of hemizygosity, ring chromosome instability and somatic mosaicism [3, 4, 7, 8]. Besides, the presence of additional chromosomal abnormalities also can be considered. In this regard, whole-genome high-resolution cytogenetic analysis proves to be indispensable for comprehensive diagnosis, accurate genetic counseling and clinical management of the families.

In this report, we characterize a patient who apart from r(18) clinical manifestations exhibited a severe congenital subaortic stenosis, a heart malformation usually not described in ring 18 cases. Array-based comparative genomic hybridization (aCGH) analysis allowed us to refine the boundaries of r(18) terminal deletions and thus to define a hemizygous region responsible for the main phenotypic features. We also revealed an interstitial heterozygous microdeletion encompassing a cardiac gene, which might have an additional impact on the heart malformation of the patient.

## Case presentation

### Clinical report

The proband was born at term as the first child of healthy non-consanguineous parents of Russian origin. At birth the patient presented multiple malformations typical for r(18) syndrome, namely microcephaly, low-set deformed ears, broad flat nose, a carp-shaped mouth, gothic palate, hypertelorism, broad and short great toe and syndactyly. At 3 years old, a subaortic stenosis and mental retardation were diagnosed. Two years later, at 5 years, subaortic stenosis was surgically corrected followed by a repeated operation and Ross-Konno procedure at the age of 13 and balloon angioplasty at the age of 18 years.

### Results

**Fig. 1:**
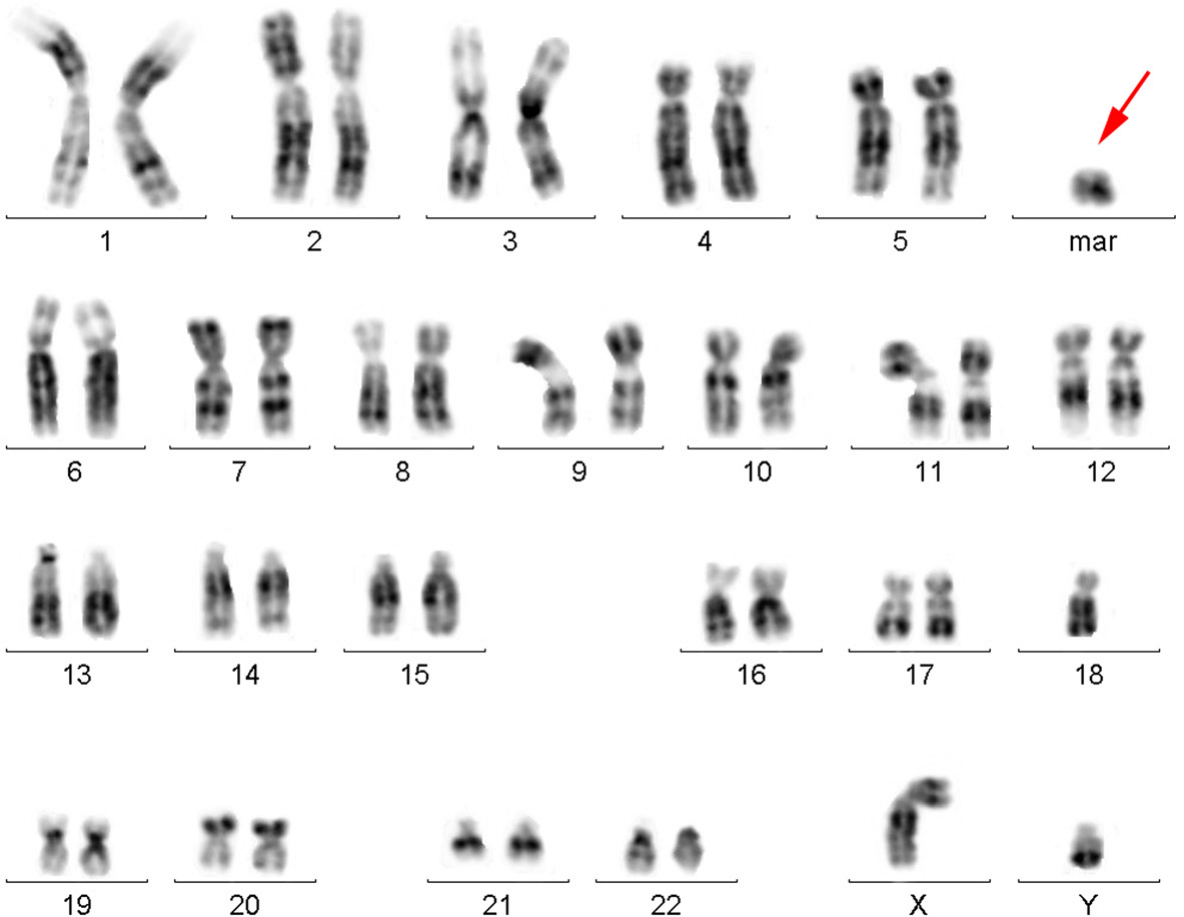
Standard cytogenetic analysis of the patient. GTG-banded karyotype showing the presence of ring chromosome 18 (karyotype 46, XY, 18(r)). Red arrow points to the ring chromosome (mar: marker)

**Fig. 2:**
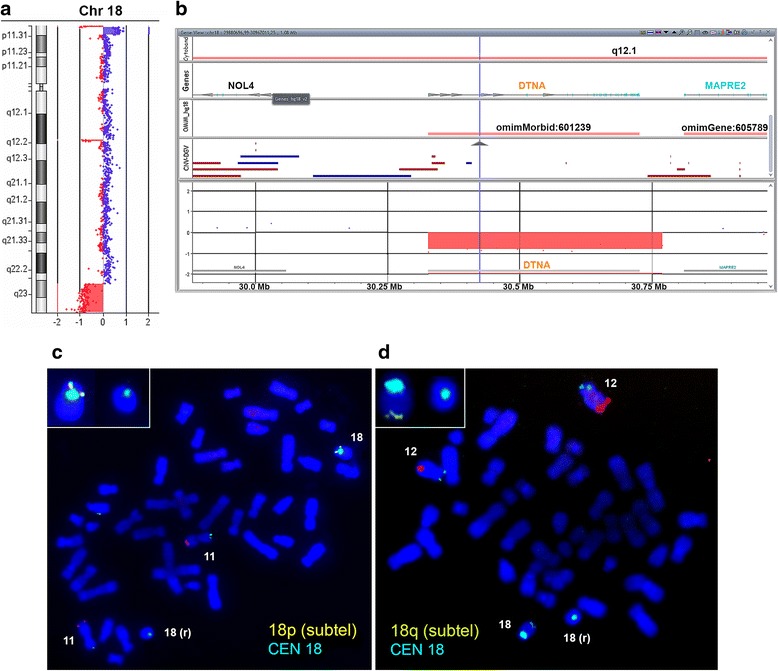
High-resolution molecular cytogenetic analysis of the patient. **a**, **b** Comparative genomic hybridization using Agilent 60 K microarray. **a** The chromosome 18 view; oligonucleotides with log2 ratio ~ −1 (red dots, red rectangles) indicate deleted regions; oligonucleotides with log2 ratio ~ +0.6 (blue dots, blue rectangle) indicate a duplicated region. The boundaries of terminal deletions, a duplication and an interstitial deletion (18q12.1) were defined with high resolution. **b** The enlarged 18q12.1 region with imported DGV and OMIM databases tracks and genes annotations. The data illustrate the presence of *DTNA* deletion. **c**, **d** Fluorescent *in situ* hybridization (FISH) on metaphases from cultured peripheral blood lymphocytes. FISH with probes to centromeric (*aqua*) and subtelomic (*yellow*) regions of chromosome 18 (ToTelVysion Probe Kit, Abbott/Vysis; the probes to subtelomeric regions of chromosomes 11 and 12 (p – green, q – red) are added in the hybridization mix by the manufacturer). Inserts show the enlarged view of the normal and the ring chromosome 18. The analysis confirmed the deletion of both terminal regions on r(18)

The standard cytogenetic analysis showed the presence of r(18) in proband’s karyotype (46, XY, r(18)) (Fig. 1, Additional file 1) with the ring chromosome being revealed in all analyzed metaphase plates. The deletion of both 18p and 18q terminal regions of r(18) was unambiguously confirmed by FISH with probes marking subtelomeric regions of chromosome 18 (Fig. 2c, d). That is, hybridization signals were specifically detected on a normal chromosome 18 homolog but were not revealed on r(18).

Array-CGH analysis showed that 18p terminal deletion corresponded to the 18p11.32 cytoband (Fig. 2a) and spanned 305 kb (chr18: 132096–437282 bp, hg18 build). The 18q terminal deletion was assigned to the 18q22.3–q23 chromosomal region (Fig. 2a) with length about 7.5 Mb (chr18: 68533620–76083117 bp, hg18 build) that involves many annotated genes including OMIM morbid genes *TSHZ1* and *CTDP1*. Additionally, a duplication adjoining the breakpoint of the 18p deletion and spanning approximately 2 Mb (chr18: 467510–2480365 bp, hg18 build) was detected. Apart from the terminal imbalances, an interstitial heterozygous deletion of 442 kb in size (chr18: 30327060–30769230 bp, hg18 build) was found on chromosome 18 (Fig. 2a, b). The deletion involved only the *DTNA* gene (http://www.ncbi.nlm.nih.gov/gene, ID: 1837; OMIM 601239) and encompassed the whole gene sequence. The deletion was verified by qPCR (Additional file 2). As parental biomaterial was not available, we could not determine the origin of the deletion.

### Discussion

Here we report on one more case of ring chromosome 18. Whole-genomic microarray-CGH analysis allowed to refine the r(18) deletion breakpoints with high resolution and thus to define the content of hemizygous genes. The 18p deletion involved the very terminal chromosomal region and affected the entire *USP14* and *THOC1* genes and the most part of *COLEC12* gene. These genes are not assigned to morbid ones, and, according to currently available data, they do not belong to critical regions and have not been discussed as putative causative genes for r(18) severe phenotypic features.

An additional relatively large duplication at the site of the 18p deletion breakpoint was revealed, which agrees with the available data on high-resolution molecular-cytogenetic analysis of patients with ring 18. That is, about 20 % of ring chromosome cases, including r(18), have been shown to be accompanied by duplication of the regions bordering the terminal deletion breakpoints [3, 9]. Together with hemizygosity extent, ring chromosome instability and somatic mosaicism, such duplications are regarded as a cause of r(18) phenotypic heterogeneity. In the case presented here, the duplication spanned about 2 Mb and involved eight protein-coding genes, including OMIM genes *COLEC12, CETN1, TYMS, ENOSF1, YES1* and *ADCYAP1*. In DECIPHER database, several CNV (copy number variant) gains similar in position, length and gene content have been described (Decipher IDs 289491, 252149, 253424) with their pathogenicity and clinical significance being largely uncertain. The phenotypes annotated for these cases have included ataxia, oculomotor apraxia, and proportionate short stature. The association between increased dosage of the above-mentioned genes and congenital craniofacial and/or cardiac defects has not been described. A patient exhibiting *ADCYAP1* (or, *PACAP*) overexpression, as a consequence of a partial trisomy 18p, has suffered from severe mental retardation [10]. This is a reasonable link, since *PACAP* is known to encode a neuropeptide that plays a role in regulation of neuronal development, differentiation and survival [11–13]. Taking all of this into account, the revealed 18p duplication does not seem to be responsible for the severe congenital defects (facial dysmorphia and the heart defect) presented by the patient but could contribute to the individual phenotype, in particular, to mental delay.

The 18q terminal deleted region was quite extended and encompassed 27 RefSeq protein coding genes including several OMIM morbid genes known to be associated with a number of particular congenital dysmorphia and intellectual disability, which correlates with the patient’s clinical manifestations. That is, heterozygous disruption of *TSHZ1* (teashirt zinc finger homeobox 1) gene was found to be responsible for congenital aural atresia phenotype in mice [14] and humans [15, 16]. Additionally, deletion of this gene has been implicated in congenital foot malformations/deformations [15, 16] as well as in cleft lip and palate formation [14, 16]. Finally, together with *ZADH2* gene, *TSHZ1* belongs to a critical region associated with mood disorder in people with 18q distal deletion [17]. Mutations in *CTDP1* gene were identified in Roma/Gypsy population as a cause of Congenital Cataracts Facial Dysmorphism Neuropathy (CCFDN) syndrome (OMIM 604168) characterized by anomalies of the eye, impaired physical growth, mild facial dysmorphism and a hypo/demyelinating, symmetric, distal peripheral neuropathy [18]. The loss of *SALL3*, another gene located in the 18q-deleted region, has been shown to underlie craniofacial development in mice [19]. Some other genes from the region are also known to be involved in regulation of crucial biological functions, but their implications in r(18) clinical features still need to be clarified.

On the whole, we conclude that the patient’s phenotypic picture distinctive for ring chromosome 18 cases is largely determined by haploinsufficiency as a consequence of hemizygosity of the 18q22.3–q23 region. However, it is worth noting that the patient also presented a severe congenital heart defect, namely, subaortic stenosis. At present, there is a lack of data on genotype-phenotype correlations for heart malformations in ring 18 individuals. Recently, van Trier and coauthors reported on two patients with 18q terminal deletion and complex cardiac abnormalities and compared the data with earlier published del(18q) cases [20]. As a result, the authors have suggested the most distal part of 18q as a critical overlapping region for heart malformation.

According to the published data, the cardiac anomalies that are predominantly described for r(18) and 18q deletion syndrome include pulmonary stenosis and atrial septal defects [3, 16, 20, 21]. Congenital subaortic stenosis is a heart malformation not typical for ring chromosome 18 condition. The only example of association between hypertrophic subaortic stenosis and a ring chromosome from E group, presumably r(18), was described by Wald and coauthors [22]. In that study, karyotyping was carried out on the basis of chromosome morphology and tritiated tymedin incorporation pattern, and unambiguous identification of the chromosome formed a ring was not possible. Besides, the boundaries of ring chromosome terminal deletions, as well as any additional subtle chromosomal rearrangements contributing to the clinical picture could not be examined without comprehensive cytogenetic and molecular-cytogenetic analysis.

We showed that the patient was a carrier of an interstitial deletion corresponded to whole *DTNA* gene. *DTNA* encodes α-dystrobrevin, a member of dystrophin-associated glycoprotein (DAG) complex, which is thought to provide the integrity and maintenance of sarcolemma and to be involved in muscle contraction and relaxation signaling [23–25]. The previous studies gave evidence that α-dystrobrevin includes four main structural-functional domains and directly interacts with some members of DAG complex, in particular, with dystrophin, syntrophin and sarcoglycan complex [23, 24, 26]. The current notion of the DAG complex network in muscle and its schematic illustration was provided by Nakamori and Takahashi [26]. It is now well known that abnormalities in different members of this complex cause various muscular dystrophies and cardiomyopathies. Specifically, mutations in the dystrophin gene are responsible for Duchenne (OMIM #310200) and Backer (OMIM #300376) muscular dystrophies as well as for X-linked dilated cardiomyopathy (OMIM #302045). Sequence variations in other DAG members including α-, β-, γ-, and δ- sarcoglycans, integrin α7 and laminin α2 are also found to underlie muscular dystrophies and cardiomyopathies [27–32].

Much less is known about α-dystrobrevin. According to several studies, α-dystrobrevin deficiency is also associated with congenital muscular dystrophy [26, 33–35]. At present, the data on α-dystrobrevin functioning in heart and its role in pathogenesis of cardiovascular diseases are especially limited. Mice deficient in α-dystrobrevin exhibited both skeletal and cardiac myopathies [36]. Besides, the hearts of α-DB null (*adbn−/−*) mice were shown to be highly susceptible to injury during cardiac stress [25]. In human, it has been shown that *DTNA* missense mutations were associated with left ventricular noncompaction 1 cases (OMIM #604169) - both isolated cases and/or conditions with associated congenital heart defects including coronary artery anomalies, conotruncal anomalies, ventricular and atrial septal defects, pulmonic stenosis, anomalous venous pulmonary veins, hypoplastic left heart syndrome, Ebstein’s anomaly [37–39].

With regard to *DTNA*, it proves to be especially important to focus on genotype/phenotype correlations not only in cases of single nucleotide changes but whole-gene deletion as well. There are five known α-dystrobrevin isoforms resulting from alternative splicing [23, 40, 41], therefore in case of complete gene deletion the depleted protein function can not be compensated by remaining isoforms. By now, the condition of complete α-dystrobrevin gene deletion has been modeled in mutant mice [42]. To our knowledge, just a few cases of 18q deletion involving *DTNA* have been described in human. In particular, the data on two deletions from PubMed literature [43, 44] and five deletions from the DECIPHER database [45] are available (Decipher IDs 250878, 260121, 276030, 286198, 288657). Along with severe intellectual disability and motor delay, in some of these cases the heart and great vessels’ malformation as well as hypotonia were observed. At the same time, these deletions spanned as much as 3.2–14.5 Mb in size, encompassed several morbid genes and, in some cases, were associated with additional chromosomal micro-rearrangements. For this reason, the interpretation of genotype-phenotype correlation as well as the assessment of *DTNA* deletion impact on the phenotypes is ambiguous. Here, for the first time we presented a case of the 18q12.1 deletion restricted only to *DTNA* gene in a patient with subaortic stenosis that allows to suggest that *DTNA* belongs to dosage sensitive genes and to extend our knowledge of the role of *DTNA* haploinsufficiency in congenital heart malformation.

## Conclusions

In conclusion, we report on a patient with ring chromosome 18 phenotype combined with a congenital subaortic stenosis. The results of array-CGH analysis imply that a combination of several genetic aberrations such as terminal imbalances of r(18) and 18q12.1 interstitial microdeletion might contribute to the complex phenotype. Our study draws attention to *DTNA* gene whose deficiency was possibly implicated in the described congenital heart malformation, but further careful functional studies are needed. Here we support the utility of array-CGH analysis for accurate individual diagnosis, disease prognosis and genetic counseling of patients with multiple congenital defects and non-typical clinical picture.

## Methods

Routine analysis of G-banded metaphase chromosomes at a resolution of 400 bands was performed using phytohaemagglutinin (PHA)-stimulated peripheral blood lymphocytes, with fifteen metaphase plates being analyzed. Fluorescent *in situ* hybridization (FISH) with molecular probes specific to centromeric and subtelomeric regions of chromosome 18 (ToTelVysion Probe Kit, Abbott/Vysis) was carried out on metaphase spreads of the same lymphocyte suspension.

Whole-genome DNA analysis was performed using oligonucleotide array-based CGH. As a platform, Agilent 60 K array with median probe spacing 41 kb was used (SurePrint G3 Human CGH Microarray, Agilent Technologies, Santa Clara, CA, USA). The study was performed according to Helsinki Declaration and study approval was obtained from Institutional Ethical Review Board at the Almazov Federal Medical Research Centre in St. Petersburg. Written informed consent was obtained from the patient and his parents prior to investigation. Genomic DNA was extracted from peripheral blood cells using a Puregene DNA Extraction Kit (Gentra, Qiagen, USA). The sample preparation and the hybridization procedure were carried out according to manufacturer’s recommendations. The data obtained was processed and analyzed using CytoGenomics Software (v3.0.1.1, Agilent Technologies) with imported tracks of publically available databases of normal and pathogenic human genome variants, such as Database of Genomic Variants (DGV) [46] and Online Mendelian Inheritance in Man database (OMIM) [47]. Additionally, the revealed copy number variants were compared with the data from DECIPHER [45] and pubmed [48] databases. CNVs (gains and losses) were called using an aberration detection statistical algorithm ADM-2, with a sensitivity threshold of 6.0. All genomic coordinates refer to the human reference assembly NCBI36 (hg18).

The loss of 18q12.1 region was confirmed by real-time quantitative PCR (qPCR) using SYBR Green Master Mix according to manufacturer’s recommendations (http://www.evrogen.com/). The assay was performed for *DTNA* gene sequence (exon 6; NG_009201.1) with the following primers designed using NCBI Primer Blast tool [49]: F 5′-TTGCGGGAAAATGCTCTGAAC-3′; R 5′- TAAGGAGGAGGCTGATGGACT-3′. The quantity assessment of the target sequence was carried out relative to a normal control DNA. The relative copy number was evaluated using the comparative ΔΔCt method with the housekeeping gene glyceraldehyde-3-phosphate dehydrogenase (*GAPDH*) being used for normalization.

The structural variants were submitted to the DGV archive [50] with accession number estd226.

## Ethics, consent and permissions

Written informed consent was obtained from the patient and his parent prior to investigation for publication of this Case report and any accompanying images. A copy of the written consent is available for review by the Editor-in-Chief of this journal.

## Additional files

Additional file 1:
**Standard cytogenetic analysis of the patient (additional material).** Two GTG-banded metaphase plates showing the presence of ring chromosome 18 (karyotype 46, XY, 18(r)). Red arrows point to a normal chromosome homolog 18 and to a ring chromosome r(18). (PNG 437 kb) Additional file 2:
**Confirmation of** ***DTNA*** **deletion in the patient using quantitative real-time PCR analysis (qPCR).** Description of data: qPCR data revealed one copy of the *DTN*A gene (18q12.1) in a patient DNA sample as compared to two copies of the gene in a normal control DNA sample. The data was normalized against *GAPDH* gene using the comparative ΔΔCt method. RQ (relative quantity) value is presented along the vertical axis. Each reaction was reproduced (repeated) in triplicate for both DNA samples (patient and control) and both genes (*DTNA* and *GAPDH)*. The series of four ten-fold dilutions were included into analysis with the starting amount of DNA ~ 1 ng. The results obtained for one of the dilutions are depicted in the figure; for the rest dilutions, the ratio of quantity values between test and control samples was the same. (PNG 8 kb)

## Abbreviations

aCGH: array-based comparative genomic hybridization
CNV: copy number variant
DAG: dystrophin-associated glycoprotein complex
DECIPHER: database of chromosomal imbalance and phenotype in humans using ensembl resources
DGV: database of genomic variants
FISH: fluorescent *in situ* hybridization
OMIM: online mendelian inheritance in man database
PHA: phytohaemagglutinin
