# Supplementary figures and images for: Ring chromosome 18 in combination with 18q12.1 (DTNA) interstitial microdeletion in a patient with multiple congenital defects
Source: Mol Cytogenet. 2016 Feb 18;9:18. doi: 10.1186/s13039-016-0229-9 (PMC4758088; doi:10.1186/s13039-016-0229-9)

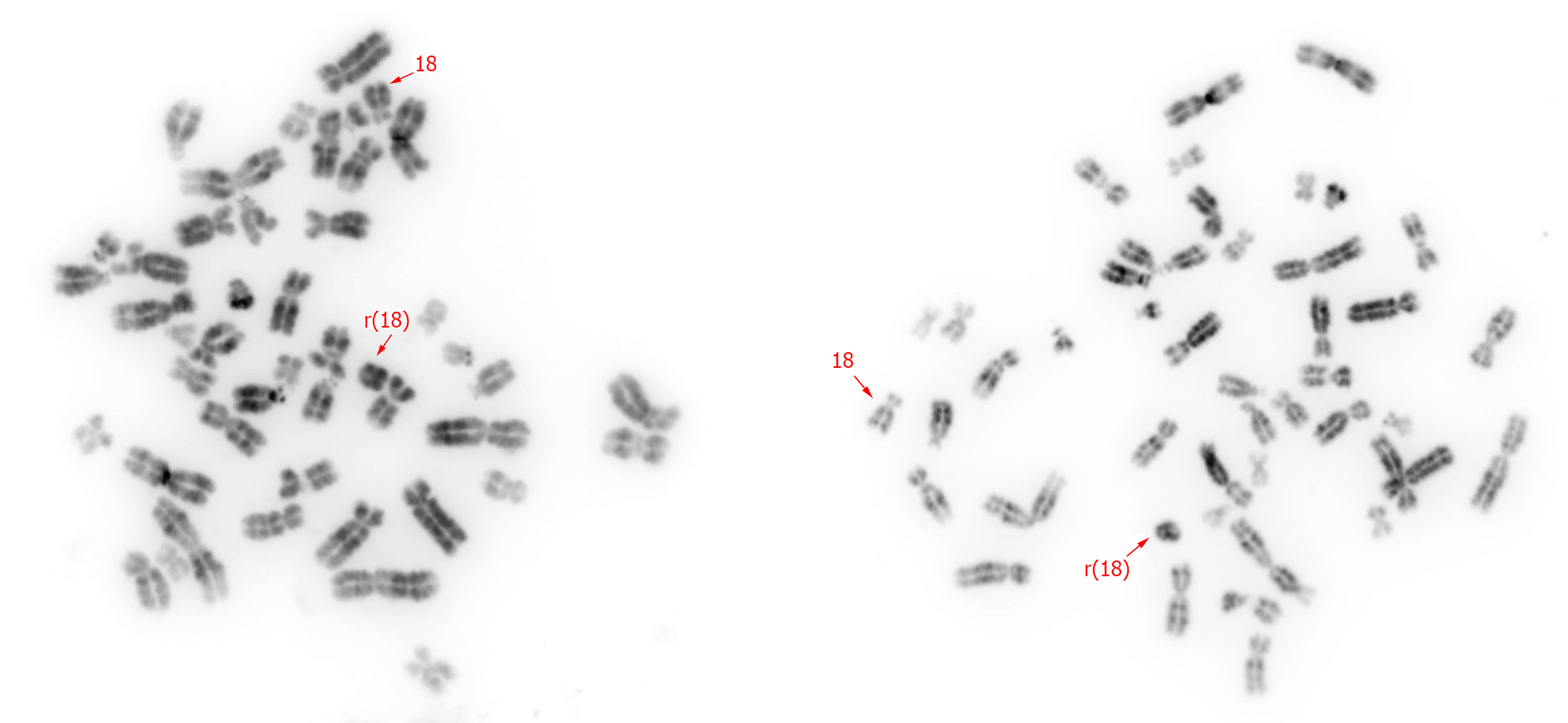

Supplement: Additional file 1: — Standard cytogenetic analysis of the patient (additional material). Two GTG-banded metaphase plates showing the presence of ring chromosome 18 (karyotype 46, XY, 18(r)). Red arrows point to a normal chromosome homolog 18 and to a ring chromosome r(18). (PNG 437 kb) [file 13039_2016_229_MOESM1_ESM.png]

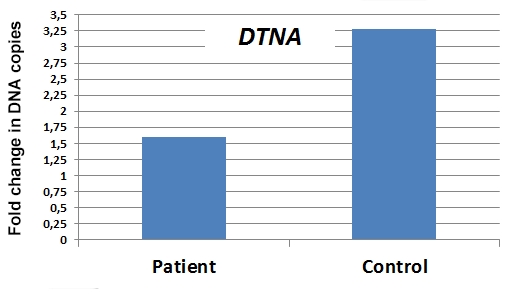

Supplement: Additional file 2: — Confirmation of DTNA deletion in the patient using quantitative real-time PCR analysis (qPCR). Description of data: qPCR data revealed one copy of the DTNA gene (18q12.1) in a patient DNA sample as compared to two copies of the gene in a normal control DNA sample. The data was normalized against GAPDH gene using the comparative ΔΔCt method. RQ (relative quantity) value is presented along the vertical axis. Each reaction was reproduced (repeated) in triplicate for both DNA samples (patient and control) and both genes (DTNA and GAPDH). The series of four ten-fold dilutions were included into analysis with the starting amount of DNA ~ 1 ng. The results obtained for one of the dilutions are depicted in the figure; for the rest dilutions, the ratio of quantity values between test and control samples was the same. (PNG 8 kb) [file 13039_2016_229_MOESM2_ESM.png]
